# Willingness to participate in weight-related research as reported by patients in PCORnet clinical data research networks

**DOI:** 10.1186/s40608-018-0187-3

**Published:** 2018-03-01

**Authors:** William J. Heerman, Wendy L. Bennett, Jennifer L. Kraschnewski, Elizabeth Nauman, Amanda E. Staiano, Kenneth A. Wallston

**Affiliations:** 1 Vanderbilt University Medical Center, 2146 Belcourt Ave, 2nd Floor, Nashville, TN 37212 USA; 2 The Johns Hopkins University School of Medicine, Baltimore, MD USA; 3 The Pennsylvania State University, College of Medicine, Hershey, PA USA; 4 Louisiana Public Health Institute, New Orleans, LA USA; 5 Pennington Biomedical Research Center, Baton Rouge, LA USA; 6 Vanderbilt University School of Nursing, Nashville, TN USA

**Keywords:** Research participation, Clinical data research network, Obesity

## Abstract

**Background:**

Since 2014 the Patient Centered Outcomes Research Institute (PCORI) has funded 13 Clinical Data Research Networks (CDRNs) around the country to support large-scale comparative effectiveness research and pragmatic clinical trials. To provide guidance for future recruitment efforts among CDRNs this study described differential willingness to participate in weight-related research by body mass index (BMI) and sociodemographic characteristics.

**Methods:**

During 2014–2016 we surveyed participants from three CDRNs including the Mid-South CDRN, REACHnet, and the PaTH Network, representing 14 medical centers. Participants were eligible if they were ≥18 years, had ≥2 weights and ≥1 height in the electronic health record. Respondents were recruited face-to-face in primary care and specialty clinics, and via email from doctors’ offices, patient registries and health systems’ patient portals. Data was collected on willingness to participate in weight-related research (four items combined into a single scale; range 4–12), BMI, and sociodemographics (age, sex, number of people in household, marital status, education level, race, and ethnicity). Adjusted ordinal regression models tested associations between participant characteristics and willingness to participate in weight-related research.

**Results:**

Among 11,624 respondents, mean BMI was 29.6 (SD 7.6) kg/m^2^. Mean willingness to participate in weight-related research was 7.1 (SD 2.5). More respondents were willing to participate in studies with lower burden: healthy lifestyles (82.2%), genetics (71.3%), medication (52.2%), and surgery (22.6%). In adjusted models, higher BMI was associated with greater willingness to participate in weight-related research (OR = 1.13) as were younger age (OR = 0.98), being a woman (OR 1.59), and college education (OR = 1.72) (all *p* < 0.001).

**Conclusions:**

Associations among BMI, age, sex, and education level with willingness to participate in weight-related research highlight the need for future research to reduce barriers for populations less willing to engage in weight-related research.

## Background

Developing evidence-based solutions to the obesity epidemic depends on the willing engagement and participation of individuals who represent a range of social, racial/ethnic, and geographic backgrounds [1]. The external validity (or generalizability) of medical evidence becomes speculative when certain groups of people are more or less likely to participate in biomedical research, limiting the applicability of research findings to potentially important population sub-groups [2, 3]. Differential willingness to participate in research is especially relevant for medical conditions, such as obesity, where under-represented minorities have a disproportionate disease burden [4]. For decades, there has been concern that minority patients may be less willing to participate in clinical trials because of distrust of the medical field [5, 6]. Recent reports show comparable, if not higher, participation rates in clinical or other health-related research for non-Hispanic, Black and Hispanic groups compared to non-Hispanic Whites, suggesting minority patients may be more willing to engage in research than previously thought [7]. Consequently, understanding differential rates of willingness to participate in *weight-related* biomedical research is an important component of developing equitable and generalizable approaches to combatting the obesity epidemic.

Since 2014 the Patient Centered Outcomes Research Institute (PCORI) has funded 13 Clinical Data Research Networks (CDRNs) around the country to support large-scale comparative effectiveness research and pragmatic clinical trials [8]. Each network consists of multiple healthcare institutions, including practices and hospitals, and each network comprises data from electronic health records for at least 1,000,000 unique patients. The CDRNs share a set of clinical data elements, and a single coordinating center can administer distributed research queries on these data networks. With such a large, nationwide data infrastructure, CDRNs hold the promise of overcoming barriers to efficiently conducting large scale clinical research through a national network capable of recruiting patients representative of the general population in the United States.

A critical component of the CDRNs’ ability to recruit for registries, cohorts and pragmatic clinical trials is the willingness of people to participate in clinical research. Because of the public health burden of obesity, each CDRN created an electronically identified cohort of adult patients with data available on both height and weight (i.e, body mass index, BMI) for the purpose of conducting weight-related research. Each network surveyed a sub-sample of participants eligible for these “healthy weight cohorts” with questions about willingness to participate in future weight related research.

Few prior studies have examined patterns of participation in obesity-related clinical trials [9, 10]. Understanding the willingness to participate in weight-related research of potential research participants from each CDRN is relevant for both future recruitment efforts within these large networks and will also allow for more generalizable conclusions that can inform recruitment approaches for obesity research. Specifically, understanding the potential determinants of and sociodemographic variation in people’s willingness to participate in *weight-related* research could help improve efforts to recruit and retain samples that are representative of the population most affected by obesity-related conditions. Here, we report survey results from three CDRNs—the Mid-South CDRN, Research Action for Health Network (REACHnet), and the PaTH Network—to 1) describe the willingness of potential research participants to enroll in various types of weight-related research and 2) understand potential determinants of willingness to participate in weight-related research by testing associations between respondents’ characteristics (i.e., demographics, and body mass index) and their self-reported willingness to participate in weight-related research.

## Methods

### Setting and population

Participants were recruited from three CDRNs, including the Mid-South CDRN, REACHnet, and the PaTH Network. The Mid-South CDRN integrates a clinical data infrastructure across the United States, consisting of: (1) Vanderbilt University Medical Center (VUMC) partnering with Meharry Medical College, (2) the Vanderbilt Healthcare Affiliated Network (VHAN), (3) Greenway Health, and (4) the Carolinas Collaborative, a consortium of 4 academic health systems and multiple community health systems across North Carolina and South Carolina [11]. REACHnet is a partnership among Louisiana Public Health Institute and five health systems in Louisiana and Texas: (1) Ochsner Health System, (2) Tulane Medical Center, (3) Louisiana State University Health Care Services Division and Health Sciences Center, (4) Partnership for Achieving Total Health, a network of community-based health centers in the Greater New Orleans area, and (5) Baylor Scott & White Health. The PaTH Network is a collaboration between (1) the University of Pittsburgh, (2) University of Pittsburgh Medical Center, (3) Penn State Milton S. Hershey Medical Center, (4) Penn State College of Medicine, (5) Lewis Katz School of Medicine at Temple University, (6) Temple Health, (7) Johns Hopkins University, (8) Johns Hopkins Health System, (9) Geisinger Health System, and (10) the University of Utah. Data for these analyses were collected from select sites at each of the CDRNs, including Vanderbilt University Medical Center, VHAN, Greenway, Ochsner Health system, EXCElth, Access Health Louisiana, University of Pittsburgh, Pittsburgh Medical Center, Penn State Milton S. Hershey Medical Center, Penn State College of Medicine, Lewis Katz School of Medicine at Temple University, Temple Health, Johns Hopkins University, and Johns Hopkins Health System.

Eligibility criteria (Appendix A) were determined from structured data available in the electronic medical record and varied slightly by CDRN. A sample of eligible patients (*N* = 113,563) was recruited to complete an online or in-person survey, which included assessment of demographic information, psychosocial determinants of health, health literacy, [12] health behaviors, and willingness to participate in subsequent research. Respondents were recruited face-to-face recruitment in primary care and specialty clinics (including bariatric surgery clinics) and electronically via email from doctors’ offices, patient registries and health systems’ patient portals. All surveys were conducted in English for feasibility reasons. Each participant agreed to an electronic consent prior to participating in the survey. Institutional Review Boards at each of the CDRNs approved this study, including the IRB at Vanderbilt University Medical Center, Ochsner Health System, Tulane University, and Johns Hopkins. Survey data were aggregated across CDRNs through the sharing of de-identified datasets.

### Survey data

Sociodemographic characteristics were self-reported and included age, sex, number of people in the household, marital status, education level, race, and ethnicity. Participants self-reported height and weight, from which body mass index (BMI) was calculated. While BMI self-report commonly has been used in the literature, [13, 14] we also confirmed that these self-reported numbers had a high correlation (ρ = 0.92) with the most recent electronic health record measurement of BMI, which was available on 7833 respondents (67%).

The survey included four items about willingness to participate in weight-related research (Table 1). Each item had three response options: 1-Not Interested; 2-Somewhat Interested; 3-Very Interested. Principal component factor analysis shows that the four items loaded to a single scale (Cronbach’s alpha = 0.79) with a range of 4–12, with higher numbers indicating greater interest in participating in weight-related research.

**Table 1:** Distribution of Willingness to Participate in Four Types of Weight-Related Research (*N* = 11,624)

INSTRUCTIONS: We would like to know how much interest you would have if someone asked you to participate in different kinds of research projects. Pick your level of interest…	Not Interested	Somewhat Interested	Very Interested
A study about weight control that focuses on working on your diet or how active you are	17.8%	39.1%	43.1%
A study that uses medicines to help control weight	47.9%	26.8%	25.4%
A study about weight control that tries to understand the genetics of obesity and would require a blood sample from you	28.7%	34.8%	36.5%
A study about weight control that requires surgery	77.4%	13.6%	9.0%

The survey included seven items about willingness to participate in medical research studies (involving completing surveys, taking medications, giving blood samples, staying overnight in a hospital, etc.) that did not specify research pertaining to weight-related behavior or status. Each item had three response options: 1-Not Interested; 2-Somewhat Interested; 3-Very Interested. Scale analysis showed this 7-item measure was internally consistent (Cronbach’s alpha = 0.85) with a range of 7–21. Higher scores signify a greater willingness to participate in medical research. We included these items as a covariate in the present analysis to account for 1) any potential social desirability bias in the willingness to participate in weight-related research scale, which would also be present in this scale, and 2) to control for a more general willingness to participate in research. The Pearson correlation between the two measures of willingness to participate in research is 0.50 (*p* < 0.001), indicating that the two scales are measuring similar but distinct constructs.

### Statistical analysis

Demographic characteristics were summarized using mean and standard deviation (SD) for continuous variables and using percentages for categorical variables. Race and ethnicity were recoded into 4 mutually exclusive categories (White, non-Hispanic; Black, non-Hispanic; Hispanic; and Other, non-Hispanic).

The primary outcome was the composite weight-related willingness scale. We first evaluate unadjusted associations by presenting the mean (SD) willingness to participate in weight-related research score based on BMI and sociodemographics. To determine if BMI and other sociodemographic characteristics were associated with willingness to participate in weight-related research, we conducted ordinal regression models with the combined scale score from the four weight-related willingness items as the dependent variable, controlling for study site and general willingness to participate in research. We first ran a model with BMI as a continuous variable and then repeated the regression with BMI as a categorical variable (underweight: BMI < 18 kg/m^2^; normal weight: BMI 25–30 kg/m^2^; obese: BMI 30–35 kg/m^2^; morbidly obese: BMI > 35 kg/m^2^).

To determine if any single type of weight-related research was driving the main associations detected in the primary analysis, we conducted separate ordinal regression models using each of the four weight-related willingness items as the dependent variable with BMI and sociodemographic characteristics (age, sex, number of people in the household, marital status, highest education level attained, and race/ethnicity) as the independent variables. All adjusted models controlled for study site and general willingness to participate in research.

All analyses were run using SPSS version 24. Participants were only included if they had complete data on the weight-related willingness scale and BMI. Fewer than 5% of participants were missing data on the other variables; group medians were substituted for missing continuous variables and the most frequent category was substituted for missing categorical variables [15]. *P*-values were two sided, and statistical significance was set at *p* < 0.05.

## Results

### Patient characteristics

Survey responses (*n* = 11,624) across the three CDRNs were combined in this analysis (10,492 from Mid-South, 894 from PaTH, and 238 from REACHnet). The response rate as defined by the Council of American Survey Research Organizations (CASRO) at each of the sites varied substantially by recruitment method used. While in-person recruitment achieved up to 94% response rate, recruitment approaches using traditional paper mail resulted in a response rate between 3% and 6%. The overall response rate at each site was as follows; Mid-South 16.4%, PaTH 2.9%, REACHnet 7.7%. Mean age was 50.89 (SD 15.87) years; 71.8% were women, and the mean number of people living in the household was 2.54 (SD 1.24). The majority of participants were married or living with a partner (69.5%) and had greater than a high school degree (85.9%). The distribution of respondents’ self-reported race/ethnicity included 83.4% non-Hispanic White, 10.9% non-Hispanic Black, 2.1% Hispanic, and 3.6% Other, non-Hispanic.

### Willingness to participate in weight-related research

**Fig. 1:**
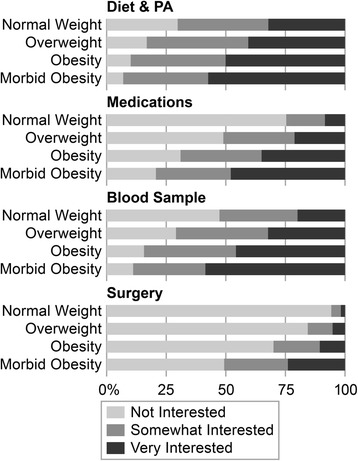
Title: Willingness to Participate in Four Types of Weight-Related Research by Weight Status. Caption: Unadjusted percentages of responses to four survey items about willingness to participate in four types of weight related research: a study that focuses on diet or physical activity, a study that uses medications, a study that requires a blood sample, or a study that requires surgery

Figure 1 displays the distributions of responses to the four items that asked about willingness to participate in future weight-related research for the entire sample and stratified by weight status. Mean score for willingness to participate in weight-related research was 7.11 (SD 2.5) on a scale from 4 (least) to 12 (greatest). More respondents were willing to participate in studies that required lower participant burden: 82.2% were somewhat or very interested in a study focusing on diet or physical activity, 71.3% were somewhat or very interested in a study focusing on genetics that required a blood sample, 52.2% were somewhat or very interested in a study that would require taking medication, but only 22.6% were somewhat or very interested in a study that would require surgery.

### Unadjusted associations between BMI and willingness to participate in weight-related research

There was an incremental increase in willingness to participate across BMI categories for studies that required the highest participant burden. Notably, 23.9% of those with morbid obesity indicated they would be very interested in a study requiring surgery compared to 10.6% of those with obesity, 5.1% of those who were overweight, and 1.6% of those who were normal or underweight. When asked about a study about weight control via diet and physical activity changes, 57.4% of those with morbid obesity indicated they would be very interested, compared to 50.0% of those with obesity, 40.4% of those who were overweight, 32.0% of those who were normal weight, and 27.7% of those who were underweight.

### Unadjusted associations between Sociodemographics and willingness to participate in weight-related research

When comparing unadjusted mean willingness to participate in weight-related research scores, younger respondents had greater willingness to participate in weight-related research: the mean age of those who were very interested in a study focusing on diet or physical activity was 48.9 (14.9) years compared to 55.1 (SD 17.4) years for those who were not interested in that same type of study. Similarly, when asked about participating in a study that would require surgery, the mean age of those very interested was 47.5 (SD 12.3) years compared to 51.7 (SD 16.5) years for those who were not interested.

Those with higher educational attainment were more likely to answer that they were interested in weight-related research. The one exception was that those with higher educational attainment were *less* likely to be willing to participate in a study requiring surgery: 6.9% of respondents who had graduated from college were very interested in a study requiring surgery, whereas 10.7% of participants with a high school degree or lower were very interested in a study requiring surgery.

### Adjusted associations between respondent characteristics and willingness to participate in weight-related research

**Fig. 2:**
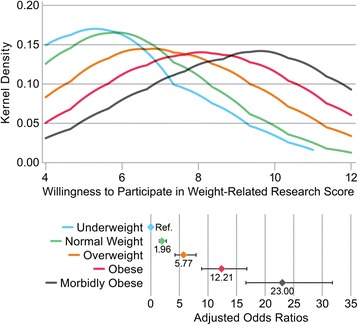
Title: Overall Willingness to Participate in Weight-Related Research by Weight Status. Caption: Distribution of Willingness to Participate in Weight-Related Research as measured by kernel density according to body mass index category (underweight: BMI < 18 kg/m^2^; normal weight: BMI 25–30 kg/m^2^; obesity: BMI 30–35 kg/m^2^; morbid obesity: BMI > 35 kg/m^2^). The inset figure shows adjusted odds ratios from multivariable ordinal regression comparing a person’s overall willingness to participate in weight-related research and weight status, controlling for age, sex, study site, number of people in the home, marital status, highest education, race/ethnicity, and their general willingness to participate in research. Odds ratios represent the increased odds for a one-point increase in willingness to participate in weight related research, which approximates a ½-standard deviation change in the scale. Error bars represent the 95% CI. All odds ratios were significant at *p* < 0.001. The referent group is underweight

Adjusted multivariable ordinal regression showed that higher BMI was associated with higher overall willingness to participate in weight-related research (β = 0.12, 95% CI (0.11, 0.13), *p* < 0.001). In addition, younger age, being a woman, being divorced/separated or married/living with a partner (vs. never married), and having higher educational attainment were all associated with higher overall willingness to participate in weight-related research in these multivariable models (all *p*-values < 0.05). We repeated the multivariable regressions using BMI category as a predictor, which demonstrated an incremental increase in willingness to participate in weight-related research for each BMI category (Fig. 2).

To determine if any of the individual items on the willingness to participate in weight-related research scale was driving potential associations, we conducted four additional multivariable ordinal regression models to assess the associations between each of the sociodemographic predictors (e.g., age, race/ethnicity) with each individual item (type of research study) on the willingness to participate in weight-related research scale (Table 2). Similar patterns of associations were observed between sociodemographic predictors and the individual items compared to the overall scale. However, even though there was a non-significant association between race/ethnicity and overall willingness to participate in weight-related research, non-Hispanic Black respondents were more likely to indicate willingness to participate in diet and physical activity studies or surgery studies but less likely to participate in medication or genetic studies compared to non-Hispanic White respondents. Further, there was no association between educational attainment and willingness to participate in a study that uses medications to help control weight.

**Table 2:** Associations (Odds Ratios) between sociodemographic variables and willingness to participate in weight-related research

	Overall scale	Diet and physical activity	Medications	Genetics	Surgery
Age	0.98‡	0.98‡	0.99‡	0.98‡	0.98‡
Sex (ref: men)	1.59‡	1.68‡	1.31‡	1.42‡	1.32‡
Body Mass Index	1.13‡	1.07‡	1.12‡	1.11‡	1.12‡
Household Number	1.02	0.98	1.05†	0.99	1.07†
Marital Status					
Never Married	(ref)	(ref)	(ref)	(ref)	(ref)
Divorced/Separated	1.49‡	1.08	1.47‡	1.46‡	1.76‡
Widowed	0.94	0.94	0.95	0.97	1.03
Married/Living with a Partner	1.24‡	1.19*	1.14*	1.25†	1.20*
Highest Education					
< High School	(ref)	(ref)	(ref)	(ref)	(ref)
High School Grad	1.44*	1.52†	1.21	1.24	0.89
Some College	1.72‡	2.07‡	1.16	1.73†	0.79
College Degree	1.53†	2.37‡	0.92	1.65†	0.57*
> College Degree	1.54†	2.58‡	0.80	1.94‡	0.47‡
Race/Ethnicity					
White, Non-Hispanic	(ref)	(ref)	(ref)	(ref)	(ref)
Black, Non-Hispanic	0.97	1.31‡	0.86*	0.73‡	1.75‡
Hispanic	1.14	1.21	0.88	1.18	1.47†
Other, Non-Hispanic	1.10	1.33†	0.88	1.13	0.83

*Note.* Five separate adjusted ordinal regression models were used, comparing sociodemographics with both overall willingness to participate in research and for each of 4 types of weight related research. Odds ratios represent the odds of a unit increase in the score presented. In addition to variables listed, regressions controlled for project site and general willingness to participate in research

*p < 0.05; †*p* < 0.01; ‡*p* < .001

## Discussion

Patients who participated in surveys from three PCORI-funded Clinical Data Research Networks are willing to participate in weight-related research, especially research on healthy lifestyles (82.2%) and genetics (71.3%), though a significant percentage indicated a willingness to participate in medication (52.2%) or surgery trials (22.6%). Our results suggest these national research networks with a shared data infrastructure have great potential to fulfill the purpose for which they were designed—to conduct large observational studies and pragmatic clinical trials among a geographically diverse and nationally representative population.

We identified greater willingness to participate in weight-related research among women, as well as participants with a higher BMI, those who were younger, and those with higher educational attainment. Additionally, participants from racial and ethnic minorities reported greater willingness to participate in some forms of weight-related research, indicating this important target population may be more likely to participate in health research than previously observed or assumed [16]. Importantly, our analyses controlled for a person’s general willingness to participate in health research, which suggests that these factors are independently associated with their willingness to participate in *weight-related* research, overcoming some concerns about potential social desirability bias. Recognizing that differential participation in research may not result from a person’s lack of willingness to participate in research, but rather may be attributable to other potential causes like time, access, or financial resources, will shape how researchers approach recruitment of populations that have been traditionally under-represented in medical research [17–20]. These findings are especially salient for ongoing efforts by the U.S. Food and Drug Administration and the National Institutes of Health to enhance recruitment efforts for traditionally under-represented minorities [21].

### Study limitations and strengths

While a significant strength of this study is the large sample size from multiple institutions across wide geographic distribution, this study does have several limitations. All of the data are self-reported, which increases the risk of misclassification and social desirability biases. The participants who said they would be willing to participate in future research had already agreed to participate in a survey, likely over-representing the general willingness to participate of all people recruited for large administrative databases. Moreover, willingness to participate in hypothetical studies may not translate into willingness to enroll in trials with specific participant burdens. As a cross-sectional analysis, this study cannot draw causal inferences particularly about respondents’ rationales for reported willingness. The overall response rates were low as sites used broad recruitment approaches including mass e-mails to large eligible patient populations, intended to test the efficacy of broad-scale recruitment techniques (i.e., unsolicited emails and postal mail) in addition to more traditional in-person techniques. Finally, the surveyed sample consisted of a majority of women and did not include a representative percentage of Hispanic/Latino respondents as the survey was only conducted in English, which could have limited the generalizability of findings.

## Conclusions

The promise of conducting large observational studies and pragmatic clinical trials from Clinical Data Research Networks requires effective recruitment of representative patient populations. Based on this large, geographically diverse sample, we conclude that a large proportion of potential research participants display a high willingness to engage in weight-related biomedical research. We also report important associations between sociodemographic characteristics (age, sex, race/ethnicity, BMI, and education level) and willingness to participate in weight-related research, providing practical guidance for where to invest future recruitment efforts, which will require identification and reduction of barriers for populations generally less willing to consider weight-related research [10]. Developing effective and sustainable evidence-based solutions for the obesity epidemic will require the involvement of a wide range of research participants from across the country. With the advent of Clinical Data Research Networks, perhaps that goal is not as far-fetched as it may have once appeared.

## Appendix A

### Eligibility Criteria for the Healthy Weight Cohort at Each Participating CDRN

Mid-South CDRN: 18 years of age or older, had at least 2 documented weights since April 30, 2009, and 1 height measurement after age 18 in the electronic health record.

PaTH CDRN: 18 years of age or older, at least 2 documented weights within the 5-year cohort time window, and at least 1 height measurement in the electronic health record. Additionally, must be proficient in English.

REACHnet CDRN: 18 years of age or older, consented to REACHnet’s *Health in Our Hands* patient registry; participants had the ability to complete the survey online in English.

## Abbreviations

CASRO: Council of American Survey Research Organizations
CDRN: Clinical Data Research Network
OR: body mass index (BMI); odds ratio
PCORI: Patient Centered Outcomes Research Institute
REACHnet: Research Action for Health Network
SD: Standard deviation
VHAN: Vanderbilt Healthcare Affiliated Network
VUMC: Vanderbilt University Medical Center
